# Lysine Acetyltransferase 2B predicts favorable prognosis and functions as anti-oncogene in cervical carcinoma

**DOI:** 10.1080/21655979.2021.1935525

**Published:** 2021-06-15

**Authors:** Lei Li, Juntao Zhang, Shuping Cao

**Affiliations:** a Department of Pathology, People's Hospital of Dongying District, Dongying City, Shandong Province, China; b Department of Gynecology, People's Hospital of Dongying District, Dongying City, Shandong Province, China

**Keywords:** Cervical cancer, kat2b, pathology, miR-93-5p

## Abstract

Lysine Acetyltransferase 2B (KAT2B) functions pivotally in regulating chromatin organization as well as function, and is a key regulator of signal transduction during development of many diseases, like tumors. This research intends to exploit expression, clinical significance as well as how KAT2B functions in cervical cancer. Our study showed that the KAT2B expression in cervical carcinoma tissues was inferior to that in normal tissues; decreased KAT2B expression was signally related to increased T staging, lymph node metastasis together with tissue differentiation; patients with high KAT2B expression had better prognosis. After knocking down KAT2B, cell proliferation diminished with decreased cell migration and invasion. Additionally, knocking down KAT2B made for increasing EMT-related proteins N-cadherin and Vimentin expression, while ZO-1 expression decreased; overexpression had the opposite effect. Dual luciferase analysis affirmed that miR-93-5p could in specifical bind to KAT2B, and thus reducing its expression and activity. KAT2B may be a new cervical tumor-suppressor gene, which is closely concerned with poor prognosis of patients, and under negative regulation by miR-93-5p.

## Graphical Abstract

**Figure:**
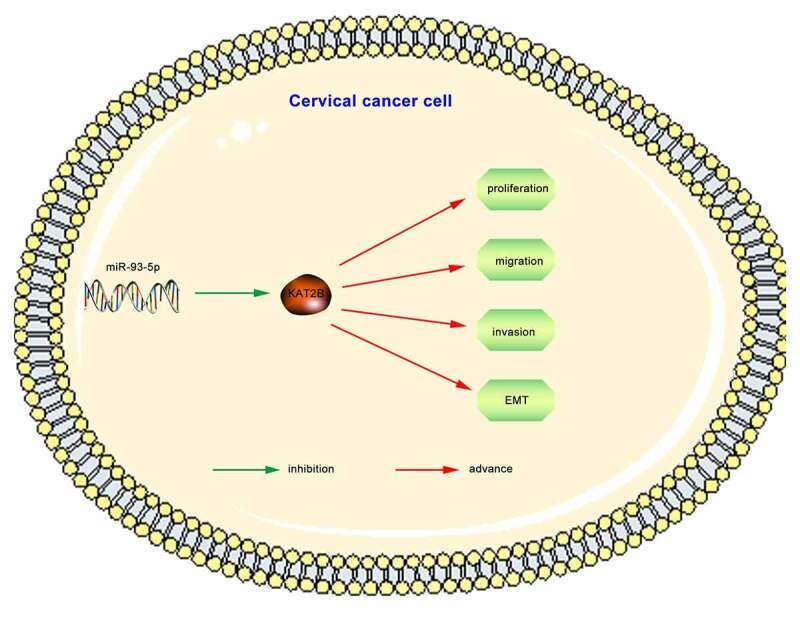

## Introduction

Cervical carcinoma is one of the most universal cancers in global females, and it is also a dominant reason for tumor-related mortality in many women in developing countries. HPV screening is a main way to diminish cervical cancer incidence [1]. The impact of early treatment on cervical cancer is better, while prognosis of patients with advanced stage is poor. Therefore, early detection and treatment are key to improving survival rate of patients with cervical cancer [2]. Although standard vaccination against human papillomavirus, rapid surgical treatment as well as regular cancer screening have signally mitigated the prevalence of cervical carcinoma, which is still one of the most fatal diseases amongst females. Therefore, it is pivotal to clarify the underlying mechanism of diseases and identify new biomarkers for the prevention and treatment of cervical cancer.

KAT as a catalyst in acetylating lysine, could impact the occurrence and development of many diseases by influencing modification of amino acid residues in proteins [3,4]. Histone acetyltransferase (HATs) belongs to KAT family, which could acetylate specific lysine in histones, and therefore functions essentially in regulating chromatin organization and function, as a key regulator of signal transduction during the occurrence of many diseases, including tumors [3,4]. KAT2B, a member of KAT family, works in acetylation and transcriptional regulation during various biological processes, by which protein encoded has the activity of acetyltransferase. In recent years, relationship between KAT2B and tumor development has been gradually revealed. Based on studies, KAT2B is highly expressed in primary alveolar rhabdomyosarcoma, and its acetylation activates PAX3-FOXO1 pathway and promotes tumorigenesis [5]. KAT2B is an essential factor in Hedgehog signaling pathway. Down-regulation of KAT2B in medulloblastoma and glioblastoma cells brings on decline in proliferation together with rise in apoptosis [6]. KAT2B linked with the occurrence of tumor cells ferroptosis in liver cancer [7]. Above findings presented that KAT2B might be the tumor biomarker and anti-cancer target in different types of tumors. However, its relationship with cervical cancer is currently unknown. Therefore, it is necessary to carry out research in this area.

Since KAT2B expression and function in cervical carcinoma have not been found, our research aims to probe into the expression, clinical significance as well as function of KAT2B in cervical cancer tissue. In current research, we firstly came up with the function and potential mechanism of KAT2B in cervical cancer, confirmed that KAT2B expression was down-regulated in cervical cancer tissues and cells, and that its overexpression was able to restrain proliferation, migration together with the invasion of cervical cancer cells, meanwhile inhibiting EMT occurrence in cervical cancer. Bioinformatics analysis identified KAT2B as a miR-93-5p target. We also confirmed that miR-93-5p had negative regulation on KAT2B expression level.

## 1. Materials and methods

### 1.1 Tissues and samples

Our team obtained frozen liquid nitrogen samples embedded in paraffin, cervical cancer tissue together with adjacent normal tissues from 52 patients in our hospital from 2013 to 2016. None of the patients received radiotherapy, chemotherapy, endocrine therapy or other tumor-specific therapies before surgery. The clinical stage was sorted via International Federation of Gynecology and Obstetrics (FIGO), including 37 cases in stage I and II and 15 cases in stage III. In accordance with histopathological evaluation, all cases were diagnosed as cervical cancer. In order to analyze the relationship between KAT2B and the survival prognosis of patients with cervical cancer, the patients were divided into high-level and low-level KAT2B groups, and the patients were followed up for up to 5 years by telephone every other week. Our reseach was approved by the ethics committee on drug clinical trial of hospital, and our team had got informed consent from our patients.

### 1.2 Immunohistochemical staining

Our team sliced wax locus containing tissue blocks, after xylene dewaxing and dehydration via gradient ethanol solution, added citric acid buffer to repair antigen under high temperature, blocked endogenous peroxidase activity with 0.3% H2O2 solution, reacted it with a blocking solution containing 10% calf serum (Gibco) for 30 minutes at room temperature, blocked nonspecific antigens, added primary antibody anti-KAT2B (1:100, Invitrogen, PA5-75,181), reacted them overnight at 4°C, and substituted PBS (Hyclone) for primary antibody as the negative control. Subsequently, added secondary antibody biotin-labeled IgG (1:1000, Abcam, ab205719), reacted them at 37°C for 1 h. Finally, adopted DAB (Beyotime) for color development, conducted HE staining, and sealed it with gelatin. Doctors in pathology department of our hospital completed scoring standard of immunohistochemistry.

### 1.3 Cell culture

We purchased human cervical cancer cell lines (C33A, HeLa, CaSki, SiHa), and normal human cervical epithelial squamous cell line H8 from Shanghai Enzyme Research Technology Co., Ltd. We placed cells in RPMI 1640 (GIBCO-BRL) medium with 10% FBS, 100 U/mL penicillin as well as 100 mg/mL streptomycin (Invitrogen); incubated them in a constant temperature incubator at 37°C, with 5% CO2 and saturate humidity.

### 1.4 Quantitative real-time PCR

We adopted TRIzol reagent (Invitrogen) to extract total RNA from tissues or cultured cells. For qPCR, we got cDNA as a result of reverse transcription of RNA through Reverse Transcription Kit (Takara). We performed qPCR analysis with Power SYBR Green (Takara). Primers we applied for qPCR were in the following:

KAT2B Forward, 5'-CGAATCGCCGTGAAGAAAGC-3' and Reverse;

5'-CTTGCAGGCGGAGTACACT-3'; GAPDH Forward;

5'-TGACAACTTTGGTATCGTGGAAGG-3' and Reverse;

5'-AGGCAGGGATGATGTTCTGGAGAG-3'miR-93-5p Forward;

GCAGCAAACTTCTGAGACAC and Reverse;

GTGCAGGGTCCGAGGTATTC;

U6 Forward, CAGCACATATACTAAAATTGGAACG and Reverse;

ACGAATTTGCGTGTCATCC.

We normalized relative KAT2B mRNA expression to GAPDH mRNA expression level, and relative miR-93-5p expression to U6 mRNA expression level; conducted qPCR on an ABI 7900HT instrument (Applied Biosystems); repeated experiment for 3 times independently.

### 1.5 RNA interference (siRNA)

In 48 well plates, we infected siRNA transfected cells with different MOI of virus as described before, removed unbound virus after 1 h, replaced it with complete media, harvested cells at different times after infection; stored them at −80°C until we collected all samples, lysed the cells via 3 freeze thaw cycles and sonication, and plated lysate dilutions on cells.

### 1.6 Cell proliferation experiment

The cell proliferation was detected as described previously [8] Selected successfully transfected cells and seeded them in 96-well plate (5000 cells/well). We chose RPMI 1640 medium as blank control group, each group with 6 replicate wells. Later our team cultured the cells for 12 h, 24 h, 48 h, 72 h, and 96 h, terminated the culture, added 10 μL CCK-8 (Sigma-Aldrich) solution and continued culture for 2 h, detected absorbance (OD) value in each well with an enzyme-linked immunoassay (Beyotime Biotechnology) at 490 nm, drew cell growth curve with time as abscissa and D_450nm_ as ordinate and repeated each experiment for 3 times.

### 1.7 Transwell assay

The Transwell experiment [9] was performed as described earlier. Our team added RPMI 1640 culture medium with 10% FBS to 24-well plate (700 μL/well), and then put Transwell chamber (8 μm aperture, BD Bioscience) into 24-well plates. If it was an invasion experiment, our team first put chamber into an empty 24-well plate, added Matrigel® (80 μL/well, 1:8) diluted with RPMI 1640 medium to chamber, and put it at 37 °C for 4–6 h; after it has solidified, our team moved chamber to a 24-well plate that has been added with RPMI 1640 medium with 10% FBS, and added transfected cells to upper chamber of Transwell (2 × 105 cells/chamber). After we cultured cells for 24 hours, took them out of chamber; immersed them in 4% paraformaldehyde solution for 10 minutes, stained them with 0.1% crystal violet solution for 30 minutes, wiped off cells in upper chamber which did not passed through cell membrane with cotton swabs, observed them under an optical microscope, and counted cells in number to present their migration together with invasion capabilities.

### 1.8 Western blot assay

After washing the cells with PBS, our team lysed them with RIPA lysis buffer (Thermo Science) containing trypsin inhibitor aprotinin, collected supernatant after high-speed centrifugation, heated it in water bath to denature its protein, quantified protein through BCA, separated protein via SDS-PAGE gel electrophoresis, transferred it to a nitrocellulose membrane (Millipore), later sealed the membrane with skimmed milk powder for 30 minutes at 37°C with 5% CO_2_, added primary antibody and Anti-GAPDH (1:3000, Abcam, ab8245) for incubation overnight at 4°C, and applied primary antibodies Anti-KAT2B (1:1000, Abcam, ab176316), Anti-E-cadherin (1:1000, Abcam, ab1416), Anti-N-cadherin (1:1000, Abcam, ab202030), Anti-Vimentin (1:1000, Abcam, ab193555) as well as Anti-ZO-1 (1: 20,000, Abcam, ab182159). After rinsing NC membrane with TBST solution, our team incubated it with a secondary antibody (Hubei Bios Biotech Co., Ltd., 1:2000) at room temperature for 1 h, and then developed it using ECL (CST), with GAPDH as an internal reference.

### 1.9 Dual-Luciferase reporter assay

The targeting relationship between KAT2B and miR-93-5p was verifies [10] as shown above. Co-transfected WT KAT2B and Mut KAT2B reporter plasmids into HEK293T cells via miR-93-5p mimic and negative control mimic. Measured luciferase activity via Dual Luciferase Reporter Assay Kit (Promega) in 24 hours after transfection. Then adopted Dual-Luciferase Reporter Assay system (Promega) to analyze. Repeated experiment for three times.

### 1.10 Data analysis

We conducted a GEO search in NCBI GEO database and obtained microarray gene expression profiles from a previous study in GEO database (GEO Accession).

https://www.ncbi.nlm.nih.gov/geo/query/acc.cgi?acc=gse26511).

We performed GSEA using GSEA v3.0 (http://broadinstitute.org/gsea/).

### 1.11 Statistical analysis

Statistical processing software in our research adopted Graphpad Prism (verse 8.0), our team presented data to be Mean ± SD. In comparison with measurement data between the two groups, and adopted *t* test, applied chi-square test for analysis on link between KAT2B expression and clinicopathological indicators of cervical carcinoma. Through Kaplan-Meier, our team drew survival curve, as well as performed log-rank test. Difference was with statistical significance with p < 0.05.

## 2. Results

### 2.1 Relative KAT2B mRNA and protein expression in cervical carcinoma

**Figure 1.**
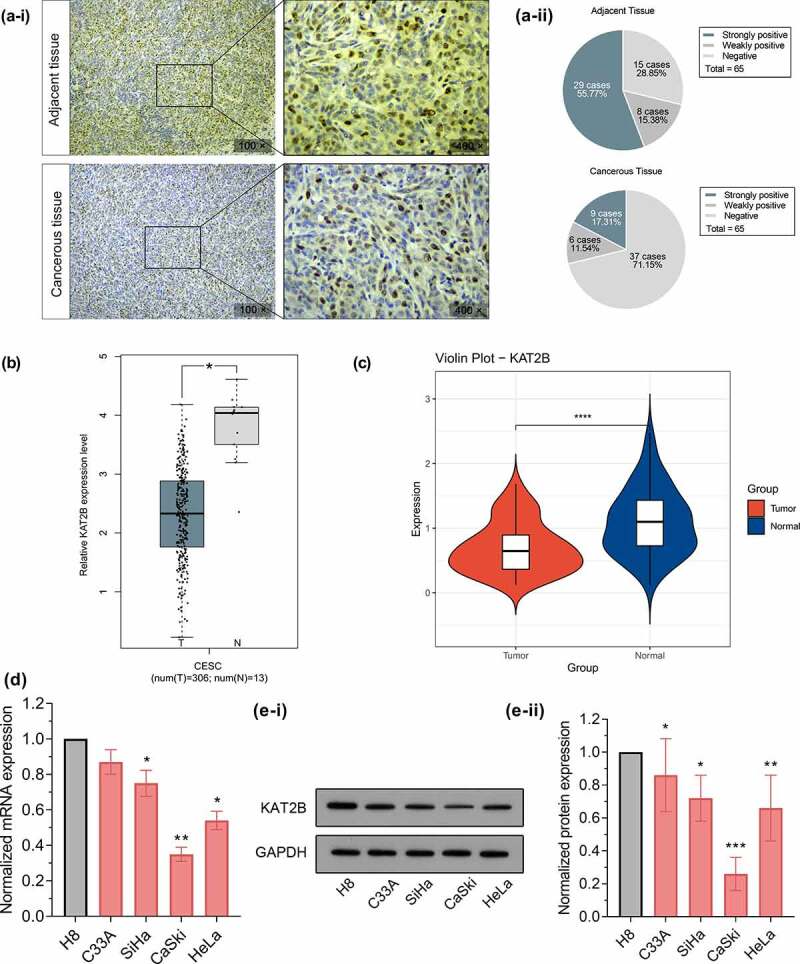
KAT2B expression in primary tumors and cell lines

With aim to initially exploit whether KAT2B act essentially in cervical cancer progression, we first applied immunohistochemistry to detect KAT2B expression in clinical samples. In adjacent cervical epithelial tissues, KAT2B protein was strongly positive for 55.77% (29/52), weakly positive for 15.38% (8/52), but negative for 28.85% (15/52). In cervical cancer tissues, KAT2B protein expression was strongly positive for 17.31% (9/52), weakly positive for 11.54% (6/52), negative for 71.15% (37/52), suggesting that KAT2B protein expression in cervical cancer tissues was signally down-regulated (Figure 1a). Based on GEPIA database (http://gepia.cancer-pku.cn/), we searched for differences in KAT2B expression in cervical cancer derived from TCGA (Figure 1b). Additionally, we adopted qPCR to determine KAT2B mRNA expression in cervical cancer and matched adjacent tissues. Similar to TCGA data, qPCR results implied that in contrast to adjacent normal tissues, KAT2B mRNA expression was signally diminished in cervical cancer tissues (Figure 1c). Subsequently, using qPCR and Western, we detected KAT2B mRNA and protein expression in normal cervical squamous cell line H8 and cervical cancer cell lines C33A, HeLa, SiHa, as well as CaSki, respectively. The results suggested that, in comparison with normal cervical squamous epithelial cells H8, KAT2B mRNA and protein expression in 4 cervical cancer cell lines were all diminished (Figure 1d and e).

(a-i) Photographs of immunohistochemistry. (a-ii) Pie charts of the immunohistochemical intensity scores of KAT2B. (b) KAT2B expression in cervical cancer from GEPIA database. (c) KAT2B mRNA expression level in 52 samples of cervical cancer tissues and paired adjacent nontumor tissues was determined by qPCR. (d) KAT2B mRNA expression level was determined by qPCR in the cervical cancer cell lines and the normal cervical squamous epithelial cell line H8. (e-i) KAT2B expression was determined in a normal cervical squamous epithelial cell line and cervical cancer cell lines by western blot analysis. (e-ii) Western blot analysis of KAT2B. *p < 0.05, **p < 0.01, ***p < 0.001.

### 2.2 KAT2B expression was related to tumor pathological indicators of patients with cervical cancer

Aiming to clarify how KAT2B functions in occurrence and development of cervical cancer and related influencing factors, we then applied aforementioned 52 cervical cancer samples for analysis on link between KAT2B expression and various pathological indicators in patients with cervical carcinoma (Table 1). In accordance with outcomes of chi-square test, low KAT2B expression in tumor tissues was obviously related to higher FIGO staging (p = 0.024), distant metastasis (p = 0.032), as well as local lymph node infiltration (p = 0.020) in patients with cervical cancer, but no obvious connection with age, tumor size as well as degree of differentiation, suggesting that low-expression KAT2B may be associated with occurrence together with metastasis of cervical cancer.

**Table 1.** Clinical characteristics of patients with cervical cancer according to KAT2B expression

Clinical features	Number of patients	KAT2B expression		χ^2^ value	*P* value
		Positive	Negative		
Age (year)				1.468	0.226
≤ 40	21	8	13		
> 40	31	7	24		
Tumor size (cm)				1.394	0.238
≥ 4	28	10	18		
< 4	24	5	19		
Differentiation				0.046	0.830
Well/moderate	30	9	21		
Poor	22	6	16		
Paplilloma virus (HPV) Infection				0.093	0.760
Yes	33	10	23		
No	19	5	14		
FIGO stage				5.125	0.024
I–II	22	10	12		
III–IV	30	5	25		
Distant metastasis				4.591	0.032
Yes	26	4	22		
No	26	11	15		
Lymph node metastasis				5.387	0.020
Yes	27	4	23		
No	25	11	14		

### 2.3 Patients with high KAT2B expression in tumors had better prognostic effect

**Figure 2.**
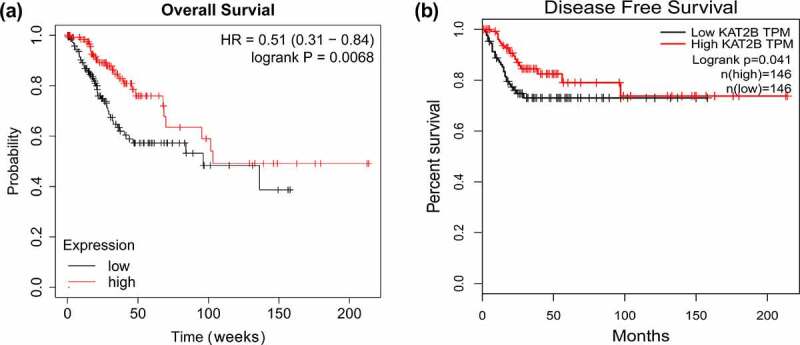
Kaplan-Meier analysis of overall survival and disease-free survival

With aim to further exploit link between KAT2B expression and long-term prognosis of patients, adopted Kaplan Meier plotter, a public plotter (http://kmplot.com/analysis/), for analysis on correlation between KAT2B mRNA expression and prognosis of patients with cervical cancer. Findings presented that overall and disease-free survival in patients with cervical cancer with low KAT2B expression were signally shorter than those in patients with high expression (Figure 2a and b), which indicated that KAT2B was a potential prognostic marker for cervical carcinoma, which might mainly work in tumor suppressor genes.

(a &b) Overall survival and disease recurrence-free survival analysis plots of KAT2B.

### 2.4 KAT2B regulated proliferation and metastasis of cervical cancer cells

**Figure 3.**
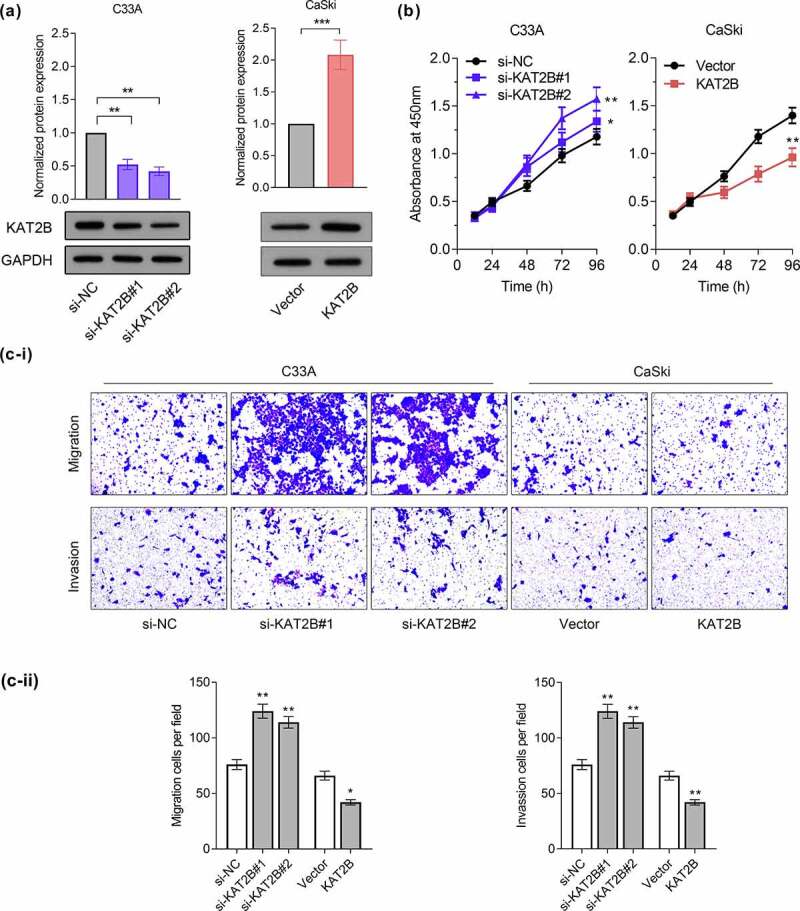
KAT2B inhibited the progression of cervical cancer *in vitro.*

With aim to probe into how KAT2B functions in regulating proliferation, migration together with invasion of cervical cancer, we successfully conducted a cell model with low and over KAT2B expression (Figure 3a). We tested proliferation ability of cervical cancer cell lines through CCK-8 experiment. Our findings suggested that knocking down KAT2B signally promoted C33A cell proliferation, while KAT2B overexpression inhibited CaSki cell proliferation (Figure 3b). Furthermore, findings of migration and invasion experiments presented that migration and invasion of C33A cells for KAT2B knockdown increased signally, while KAT2B overexpression had opposite effect (Figure 3c).

(a) The transfection efficacy was determined by qPCR. (b) Cell proliferation was measured by CCK-8. (c-i) KAT2B knockdown promoted C33A migration and invasion, KAT2B overexpression inhibited CaSki migration and invasion. (c-ii) Statistics analysis of the mean migration and invasion cell numbers as compared with the negative control (n = 3). *p < 0.05, **p < 0.01, ***p < 0.001.

### 2.5 KAT2B inhibited cell metastasis by inhibiting EMT process

**Figure 4.**
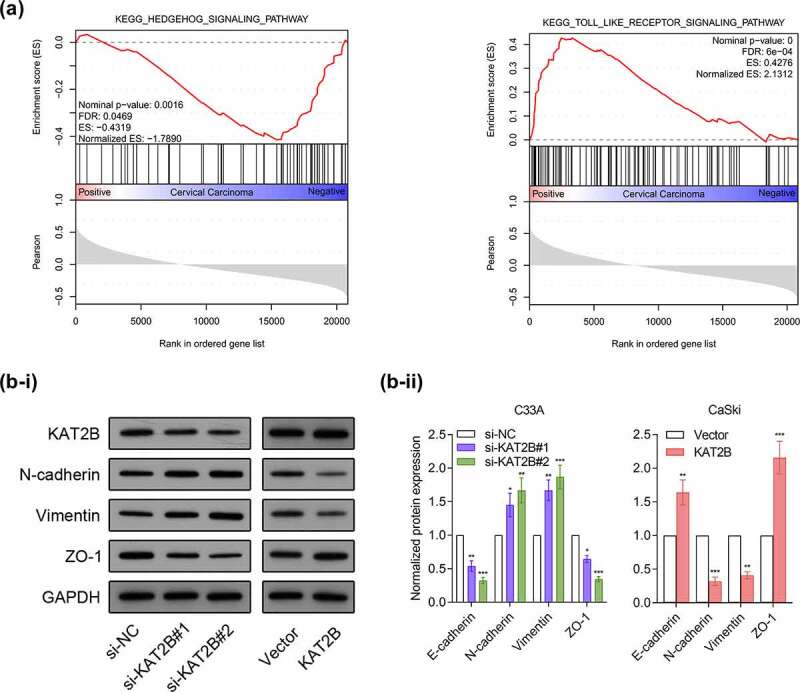
Effects of KAT2B on EMT in cervical cancer cells

We subsequently adopted GSEA3.0 for pathway analysis. Recognition results of GSEA suggested that occurrence and development of cervical cancer linked with Hedgehog signaling pathway and Toll-like receptor signaling pathway (Figure 4a). Based on research reports, Hedgehog signaling pathway together with Toll-like receptor signaling pathway are important pathways participating in regulating EMT process [11,12]. Since our experimental data indicated that KAT2B was associated with metastatic ability of cancer phenotypes, we next exploited whether KAT2B could regulate tumor metastasis markers expression. We detected three important EMT biomarkers, like N-cadherin, Vimentin as well as ZO-1 expression by Western blot. Interestingly, knocking down KAT2B could increase N-cadherin together with Vimentin expression in cervical cancer cells but decrease ZO-1 expression. However, KAT2B overexpression had opposite effect on above-mentioned marker proteins (Figure 4b), which suggested that low-expression KAT2B inhibited migration and invasion of cervical cancer cells by means of blocking EMT progression.

(a) Performance of GSEA based on GEO datasets. (b-i) Western blot assay was applied to examine EMT markers’ expression. (b-ii) Statistical analysis of western blot results. *p < 0.05, **p < 0.01, ***p < 0.001.

### 2.6 Identified KAT2B as a new miR-93-5p target

**Figure 5.**
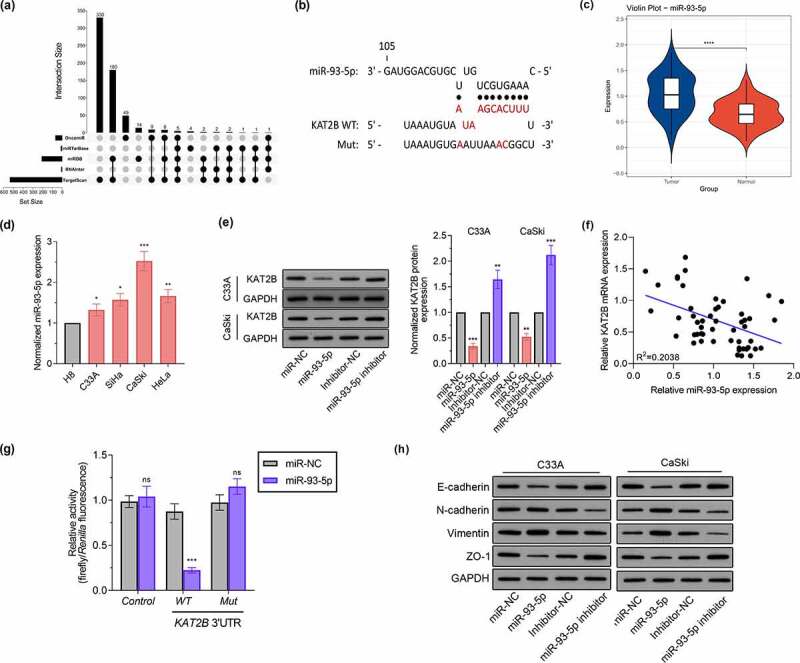
KAT2B expression was negatively regulated by miR-93-5p

KAT2B low expression mechanism in cancer tissues still needs to be resolved. Since miRNAs acts pivotally in regulating gene expression, it is of great significance to determine whether KAT2B expression is under regulation by specific miRNAs in cervical cancer. First, we searched 5 bioinformatics databases, such as OncomiR, miRTarBase, miRDB, RNAInter as well as TargetScan, to find out potential microRNAs complementary to KAT2B’s 3'-UTR (Figure 5a). Aiming to diminish false positives, solely considered candidate miRNAs which could be predicted by five means above. One was miR-93-5p, which featured a complementary site to 3'-UTR of KAT2B (Figure 5b). Existing studies have confirmed that miR-93-5p is up-regulated in cervical cancer [13,14], which we also applied qPCR to detect miR-93-5p expression in tissue samples to verify (Figure 5c). Consistent with miR-93-5p expression in cervical cancer tissues, miR-93-5p signally up-regulated in cervical cancer cell lines (Figure 5d). Therefore, we believed that low KAT2B expression was partly led by upregulated miR-93-5p. After transfecting miR-93-5p mimics or inhibitors, we observed that KAT2B expression in cervical cancer cells was inhibited by miR-93-5p overexpression. When miR-93-5p expression was inhibited, KAT2B expression level jumped (Figure 5e). we observed equal phenomenon in KAT2B and miR-93-5p expression in cervical carcinoma samples via qPCR (Figure 5f). Subsequently, we cloned WT or Mut 3'-UTR constructs of KAT2B into psi-CHECK2 vector, and co-transfected HEK293T cells with miR-93-5p mimics. Furthermore, our research presented that miR-93-5p was incapable of inhibiting luciferase activity of KAT2B in HEK293T cells (Figure 5g). These data indicated that KAT2B was a direct miR-93-5p target, and miR-93-5p expression level was negatively correlated with KAT2B. Finally, we verified miR-93-5p effect on EMT-related proteins level (Figure 5h). These findings supported that KAT2B expression in cervical carcinoma was under negative regulation by miR-93-5p.

(a) UpSet plot of miRNAs differentially expressed using five independent databases. (b) Bioinformatics database prediction of the binding site of miR-93-5p on the KAT2B 3'-UTR. (c) Relative miR-93-5p expression level was determined by qPCR in paired normal and cervical cancer tissues. (d) The expression of miR-93-5p in one normal cervical squamous epithelial cell and four cervical cancer cell lines by qPCR analyses. (e) Western blot analysis of KAT2B protein in cervical cancer cells transfected with miR-93-5p mimics or inhibitor. (f) The correlation between miR-93-5p gene expression and KAT2B expression. (g) Dual-luciferase reporter assay indicated that miR-93-5p binds to KAT2B mRNA 3'UTR directly. (h) Expression levels of EMT-associated markers in cervical cancer cell lines following transfection with miR-93-5p mimics or inhibitor were evaluated by western blot analysis. *p < 0.05, **p < 0.01, ***p < 0.001.

## 3. Discussions

In our research, we confirmed for the first time that KAT2B could inhibit cervical cancer progression. KAT2B signally down-regulated in cervical cancer tissues, and low-expression KAT2B closely linked with poor prognosis in patients. We further verified how KAT2B functioned in cervical cancer cells and confirmed that KAT2B worked in inhibiting cervical cancer. Underlying mechanism was that low-expression KAT2B inhibited migration and invasion of cervical carcinoma by inhibiting EMT, and KAT2B expression was under negative regulation by miR-93-5p.

Researches have shown that KAT2B is concerned about occurrence and development of gastric cancer, colorectal cancer, breast cancer, lung cancer, liver cancer as well as other tumors. KAT2B is able to inhibit tumorigenicity in gastric cancer cells not only in vivo but in vitro, prevent gastric cancer cells from entering S phase from G1 stage and its decrease in expression linked with poor clinical prognosis of intestinal gastric cancer [15]. KAT2B-mediated AKT1acetylation is able to enhance phosphorylation with AKT1 at threonine and serine sites, and further promotes glioblastoma cells proliferation [16]. Additionally*** [17]***, KAT2B is also capable of acetylating histone H4, inhibiting AKT signal as well as regulating GLI1/Bcl-2/Bax axis to induce apoptosis, thereby inhibiting occurrence and hepatocellular carcinoma development [14,18]. In our research, we found that low-expression KAT2B was associated with higher FIGO staging, distant metastasis, as well as local lymph node infiltration. Consistent with clinicopathological analysis, we found that KAT2B knockdown in cervical cancer cells made for proliferation and migration enhancement, while KAT2B overexpression functioned in inhibition.

Lysine acetylation, a key post-translational modification, is able to affect protein localization, stability as well as function, which is originally thought to exist solely on histones, we at present learn that tens of thousands non-histone proteins are acetylated as well. Studies have reported that TIPE1 promotes cervical carcinoma progression through inhibiting p53 acetylation, and links with poor prognosis of cervical cancer [19]. Because SP1 acetylation inhibits TERT, cervical cancer growth is inhibited [20]. Activation of β2-AR regulates p53 acetylation via up-regulating SIRT1, thereby inducing cervical cancer to produce chemotherapy resistance [21]. Lysine acetyltransferase KAT2B acetylates N-terminus of CDT1, protecting CDT1 from ubiquitination and proteasome degradation [22]. Considering that acetylation acts essentially in malignant phenotype of cervical cancer cells [23–25]. Therefore, KAT2B may also affect proliferation and metastasis of cervical cancer cells via similar mechanism. Such hypothesis needs to be further exploited in the future.

More and more evidence revealed how miRNAs important it is in gene expression regulation, and that it may involves multiple signaling pathways. miRNAs can exert positive or negative control on oncogenes or tumor suppressor genes expression to impact tumor growth. Up-regulating miR-93-5p and how it works in promoting tumor progression have been found in many tumors. For example, The circular RNA hsa_circ_0007364 through inhibiting microRNA-101-5p activated methionine adenosyltransferase IIα (MAT2A) expression to improve cervical cancer progression [26]. In addition, it was also found that T-box transcription factor TBX1, which targets microRNA-6727-5p, inhibits cervical cancer cell growth and enhances cisplatin chemosensitivity through the AKT and MAPK pathways [27]. MiR-93-5p enhances migration and invasion in head and neck squamous cell carcinoma through targeting RGMB [28]. Through down-regulating FOXA1 and up-regulating TGFB3, miR-93-5p in exosomes of tumor-associated fibroblasts enhances radiation resistance of colorectal cancer cells [29]. Our research presented that KAT2B was a direct miR-93-5p target, with negative correlation between miR-93-5p together with KAT2B expression. Interestingly, a recent study suggested that miR-93-5p promoted epithelial-mesenchymal transition of gastric carcinoma by inhibiting tumor suppressor gene ANHAK [30]. Our findings indicated that low KAT2B expression was also involved in EMT process of cervical cancer cells, revealing how dysregulated miR-93-5p expression acted in EMT process of tumor cells. Given there was high miR-93-5p expression in various tumors, which may be a potential mechanism of KAT2B down-regulation in tumors.

To sum up, this research suggested that KAT2B expression was low in not only cervical cancer tissues but cells, which was related to multiple pathological indicators and poor prognosis of patients. Inhibiting KAT2B could promote proliferation together with metastasis of cervical cancer cell line C33A, while KAT2B overexpression was capable of inhibiting proliferation as well as metastasis of cervical cancer cell line CaSki. Additionally, we confirmed that KAT2B was miR-93-5p target, which partially explained abnormally low KAT2B expression in tumors. It also revealed possible interaction between miRNAs and abnormal acetylation during cervical cancer progression.

## Highlights

1. KAT2B is under-expressed in cervical cancer.
2. KAT2B is related to the clinicopathology of cervical cancer, and patients with high-level KAT2B cervical cancer have a good prognosis.
3. Knocking down KAT2B inhibits cervical cancer cell invasion, migration and EMT.
4. MiR-93-5p binds KAT2B through targeting.
